# Effects of Age, Sex, and Social Network on Antibiotic Resistance Genes in the Gut Microbiome of Tibetan Macaques (*Macaca thibetana*)

**DOI:** 10.1002/ece3.73137

**Published:** 2026-02-20

**Authors:** Yue Ling, Dong‐Xin Yang, Ying‐Na Xia, Chuan‐Peng Bao, Fan Zhang, Xiao‐Juan Xu, Bing‐Hua Sun

**Affiliations:** ^1^ School of Resource and Environmental Engineering Anhui University Hefei China; ^2^ International Collaborative Research Center for Huangshan Biodiversity and Tibetan Macaque Behavioral Ecology Anhui University Hefei China; ^3^ School of Biology and Food Engineering Hefei Normal University Hefei China

**Keywords:** antibiotic resistance genes (ARGs), gut microbiome, metagenomics, Tibetan macaque

## Abstract

The transmission and dissemination of antibiotic resistance genes (ARGs) have increasingly drawn global attention. However, our knowledge of the antibiotic resistance gene pool in wild primates' gut microbiomes and its influencing factors remains limited. In this study, we focus on a social group of Tibetan macaques ( *Macaca thibetana* ) in Huangshan, utilizing behavioral and metagenomic data to investigate the effects of host sex, age, and social network on the ARG profiles of the gut microbiome. Our results demonstrate a high diversity of ARGs in the gut microbiota of Tibetan macaques, with multidrug, glycopeptide, and peptide resistance genes being the most prevalent. Although host age, sex, and social networks did not significantly affect the overall diversity of ARGs, these factors were significantly correlated with the relative abundance of several highly abundant ARG types, including *gryB*, *rpoB*, *macB*, *novA*, *efrA*, *patB*, *Staphylococcus aureus mupA* conferring mupirocin resistance, *RanA*, and *cdeA*. Further analysis revealed extensive interactions between gut bacteria and ARGs, with age emerging as a potentially key factor in this covariation process. These findings provide new insights into the formation and transmission mechanisms of antibiotic resistance in the gut microbiome of wildlife, particularly in social primates.

## Graphical abstract

Based on metagenomic and behavioral data from wild Tibetan macaques, this study reveals a unique gut resistome profile dominated by multidrug resistance genes. While individual factors like age, sex, and social centrality did not affect overall ARG diversity, they significantly influenced the abundance of specific, high‐prevalence ARGs. Furthermore, age emerged as a key driver of the co‐variation network between core gut bacteria and ARGs, providing new ecological insights into antibiotic resistance dynamics in wild social primates.

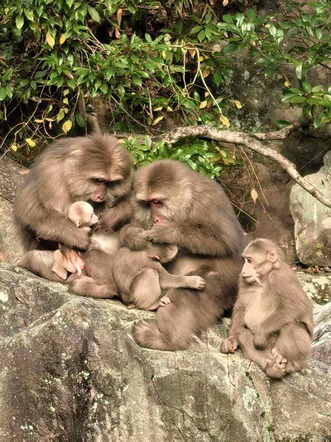

## 1 Introduction

The extensive use of antibiotics in both animal husbandry and human medicine has contributed to the emergence and dissemination of clinically relevant antibiotic resistance, which is now recognized as a pressing global public health issue (Bengtsson‐Palme et al. 2018; Zhu et al. 2019; Zhu and Penuelas 2020). The rapid evolution of drug‐resistant pathogens is progressively diminishing the efficacy of existing antibiotics, thereby undermining our capacity to treat bacterial infections effectively (Laxminarayan et al. 2013; Zhou et al. 2025). Consequently, antibiotic resistance has become a critical threat to human health worldwide and represents one of the most serious challenges of our time (Herraiz‐Carboné et al. 2021; Zhou et al. 2025). A growing body of evidence indicates that the mammalian gut serves as a significant reservoir of antibiotic resistance genes (ARGs), which can be transferred to pathogens via horizontal gene transfer (Campbell et al. 2020; Tsukayama et al. 2018). Investigating the distribution, diversity, and mechanisms underlying the formation of ARG reservoirs has become a major focus of scientific research.

The widespread dissemination of ARGs poses a serious threat to wildlife health and ecosystem stability. ARGs enter the environment through agricultural runoff, wastewater effluents, and animal feces (Zhao et al. 2023; Zhu et al. 2013), where they accumulate and are subsequently acquired by wildlife through foraging, drinking, and habitat use (Calvo‐Fernandez et al. 2025; Kauer et al. 2025). In addition to reducing therapeutic options and increasing infection risks from opportunistic resistant pathogens (Li et al. 2024), ARGs pose a systemic threat to wildlife immune function through multiple mechanisms. ARGs enrichment typically accompanies gut microbiome dysbiosis. This leads to significant reductions in key immunomodulatory metabolites such as butyrate, which in turn disrupts Treg cell differentiation and intestinal barrier integrity (Wu and Wu 2012; Zhang et al. 2019). More critically, ARGs frequently coevolve with virulence factors and mobile genetic elements, forming “superbugs” with both drug resistance and immune evasion capabilities, such as ESKAPE pathogens and carbapenem‐resistant Enterobacteriaceae in wildlife, creating the dual dilemma of untreatable infections and persistent immune exhaustion (Silva et al. 2025; Shi et al. 2025). This threat is amplified in human‐disturbed environments, where wildlife near intensive farming areas and landfills become “amplifiers” and “sentinels” for ARGs dissemination (Tallon et al. 2024). Through horizontal gene transfer networks at the wildlife‐livestock‐human interface, resistant pathogens circulate within ecosystems. During this process, they can enhance their virulence and transmissibility (Lee et al. 2022; Shi et al. 2025), ultimately compromising wildlife immune function, elevating pathogen colonization risks, and reducing survival capacity. ARGs are subsequently rereleased into environmental reservoirs via fecal shedding (Calvo‐Fernandez et al. 2025), impairing wildlife health and population dynamics—particularly in small or stressed populations—and can be transmitted through the food chain to other animals and humans. For example, *mcr‐1*‐positive *Salmonella* isolates from human patients and pork in Shanghai belonged to the same community, implicating pork as the main transmission route (Cao et al. 2022), whereas ceftiofur‐resistant *Salmonella enterica* infections in human patients were predominantly associated with chicken consumption (Otto et al. 2014). Wildlife serve as important ARG reservoirs, as demonstrated by a study of 77 individuals across 19 species in Costa Rican rescue centers where fecal shedding contaminated the environment with ARGs that may cycle back to humans and livestock (Fernandes et al. 2024). Particularly concerning are migratory wildlife, whose broad ranges facilitate cross‐regional ARG dissemination; migratory birds, for instance, can introduce resistant bacteria into new areas during seasonal movements (Li et al. 2024), expanding the geographic spread of antibiotic resistance.

As components of natural ecosystems, nonhuman primates (NHPs) are evolutionarily the closest relatives to humans. Their gut microbiomes exhibit significant functional similarities to those of humans in critical areas such as nutrient metabolism and immune regulation, making NHPs ideal models for investigating the ecological and health impacts of ARGs (Guo et al. 2023). Studying the distribution patterns and formation mechanisms of gut ARGs in primates not only aids in predicting human exposure risks but also provides cross‐species evidence within the “One Health” framework. For instance, a study analyzing metagenomic sequencing data from 131 individuals across five NHP species from diverse regions and lifestyles revealed that the composition and diversity of gut microbiota ARGs were shaped by the combined influence of host habitat and the level of human disturbance (Huang 2022). Another study on the Crab‐eating macaque ( *Macaca fascicularis* ) detected all ARG types previously identified in humans, with the composition and abundance of ARGs in their gut microbiota strongly influenced by dietary changes (Li et al. 2018).

Current research on ARGs predominantly focuses on humans, livestock, and model mice, whereas studies on nonhuman primates (NHPs) remain relatively limited. To date, NHP‐based ARGs investigations have centered mainly on comparisons between captive and wild populations (Jia et al. 2022), geographic variation in ARGs distribution (Huang 2022), and the influence of dietary factors on the gut resistome (Li et al. 2018). Existing evidence indicates that the gut microbiota of primates in social groups is modulated by individual and social factors such as host sex, age, and social networks (Guo et al. 2023; Sang et al. 2022). Since the gut microbiome constitutes a major reservoir of ARGs, its diversity and compositional structure strongly influence the types and abundance of resistance genes (Zhu et al. 2021). Therefore, individual and social factors may be potential and significant factors influencing the acquisition, persistence, and transmission of ARGs in the gut microbiota of NHP within social environments (Yuan and Chen 2020), but there is still a lack of relevant research evidence.

The Tibetan macaque ( *Macaca thibetana* ) is a primate endemic to China, belonging to the genus *Macaca* within the family Cercopithecidae (order Primates). The YA1 group of the macaques, inhabiting the Fuxi Wild Monkey Valley in Mt. Huangshan, has been continuously studied for over 40 years. To date, research on the gut microbiota of individuals within this social group has primarily focused on bacterial and fungal microbiomes (Sun et al. 2021; Xia et al. 2022). However, the composition and abundance of ARGs in their gut microbiome, the dynamic interplay between ARGs and gut microbiota, and the influence of various biological and social factors on ARG profiles remain largely unexplored. In this study, we employ metagenomic sequencing of fecal samples to characterize the gut microbiome and resistome of wild Tibetan macaques in Huangshan. Our specific objectives are: (1) to profile the composition and abundance of ARGs in the gut microbiome of Tibetan macaques; (2) to evaluate the effects of host sex, age, and social networks on the gut resistome; and (3) to analyze the interactive relationships between gut bacteria and ARGs in this species.

## 2 Materials and Methods

### 2.1 Sample Collection and Behavioral Data Collection

**FIGURE 1:**
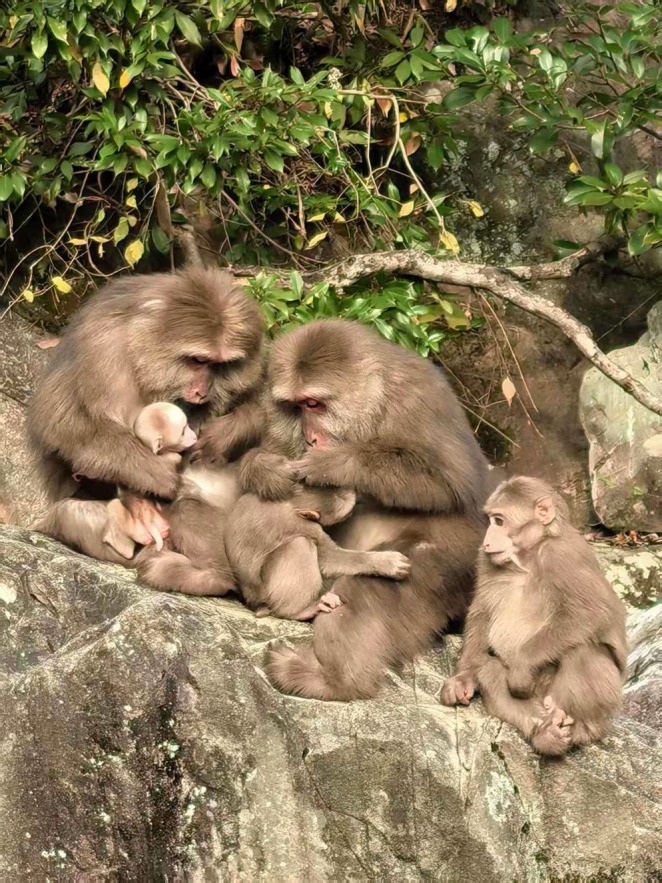
Tibetan macaques at Yulin Pit, YA1 group (Photo credit: Fang Jie).

Since 1983, our research team has conducted continuous behavioral monitoring and ecological study of the Yulin Pit YA1 group in the Fuxi Wild Monkey Valley (30°29′ N, 118°10′ E) in Huangshan, Anhui Province (Figure 1), accumulating over four decades of systematic observation. All individuals are well‐habituated and uniquely identifiable, with established genealogical relationships. The group receives daily provisioning of corn three times per day at fixed locations, accounting for approximately one‐third of their total dietary intake; the remainder consists of naturally available wild flora. The troop primarily inhabits forested areas, including for resting, foraging, social interaction, and overnight stays, and they regularly visit provisioning sites. To minimize potential seasonal effects, fresh fecal samples were collected from identified individuals of the YA1 group between July and August 2024.

In this study, fecal samples were collected using a noninvasive sampling approach. Through continuous tracking and observation of the target group, fresh fecal specimens were obtained during their daily movement, with strict adherence to the principle of one sample per individual. To minimize environmental contamination, collectors wore masks throughout the sampling process. Upon defecation by a target individual, the outer layer of the feces that had been in contact with the ground was promptly removed using sterile gloves and a disinfected sampling stick. The remaining inner portion of the sample was then immediately transferred into a sterile cryovial. Each tube was clearly labeled on both the cap and side with relevant sample information (e.g., individual identity, collection time, and age) before being rapidly stored in a liquid nitrogen container. A total of 21 fecal samples from adult individuals were included in the final analysis; all females sampled were nonpregnant and nonlactating, ruling out immediate reproductive‐state effects. Detailed sample metadata are provided in Table S1.

During the study period (July–August 2024), the monkey troop was tracked and observed daily from 08:00 to 17:00 using the focal animal sampling method. Target individuals were randomly selected using Excel‐generated sequences, and all behaviors were recorded over a 10‐min sampling period. Behaviors were recorded in real time using a Sony digital voice recorder (SONY China, Beijing, China). Among all the behaviors, we focused on observing and recording the grooming behavior, which was defined as an individual using fingers or palms to part and comb through the fur of another, occasionally picking small particles from the fur or skin and placing them into its mouth to chew (Zhang et al. 2022). A total of 11 rounds of behavioral data collection were completed, resulting in a cumulative sampling duration of 2310 min (138,600 s).

This study was approved by the Animal Care and Use Committee of the Anhui Provincial Zoological Society (Permit No. BH20221103). All procedures were conducted in accordance with the relevant guidelines and regulations.

### 2.2 Metagenomic Analysis, Taxonomic Annotation, Assembly, and Functional Prediction

Genomic DNA was extracted from fecal samples using the FastPure Stool DNA Isolation Kit (Magnetic bead; MJYH, Shanghai, China). DNA concentration and purity were quantified, and integrity was verified by 1% agarose gel electrophoresis. The DNA was then fragmented using a Covaris M220 system (Gene Company, China), and fragments of approximately 350 bp were selected for paired‐end library construction. Metagenomic sequencing was conducted on the Illumina NovaSeq X Plus platform (Illumina, USA) at Shanghai Majorbio Bio‐pharm Technology Co. Ltd. Following sequencing, raw reads were processed using fastp (Chen et al. 2018, version 0.20.0) to perform stringent quality control and filtering, retaining only high‐quality reads for downstream analyses. To minimize host‐derived contamination, the quality‐filtered data were aligned against the Tibetan macaque reference genome (GCA_003339765.3) using BWA (Li and Durbin 2009, version 0.7.17), and host sequences were removed prior to subsequent assembly and annotation.

High‐quality sequencing reads were assembled de novo using MEGAHIT (Li, Liu, et al. 2015, version 1.1.2), retaining contigs with lengths ≥ 300 bp for subsequent analysis. Open Reading Frames (ORFs) were predicted from the assembled contigs using Prodigal (Hyatt et al. 2010, version 2.6.3). Predicted genes with nucleotide lengths ≥ 100 bp were translated into amino acid sequences. All gene sequences across samples were clustered with CD‐HIT (Fu et al. 2012 version 4.7) using thresholds of 90% sequence identity and 90% coverage. The longest sequence within each cluster was designated as the representative sequence to construct a nonredundant gene catalog. High‐quality reads from each sample were then aligned to this catalog using SOAPaligner (Li et al. 2008, version soap2.21 release) with 95% identity, and gene abundance was estimated based on read coverage.

The amino acid sequences from the nonredundant gene catalog were aligned against the NR and CARD databases using Diamond software (Buchfink et al. 2015, version 2.0.13) under BLASTP parameters with an e‐value cutoff of 1e^−5^. Taxonomic annotation was assigned based on the taxonomy information associated with the NR database, and the abundance of each taxonomic group was calculated by aggregating the abundances of all genes assigned to it. Similarly, antibiotic resistance function annotations were retrieved from the CARD database, and the abundance of each ARG was determined by summing the abundances of its corresponding gene sequences. Antibiotic resistance genes were annotated using the Antibiotic Resistance Ontology (ARO), a standardized nomenclature system that assigns unique identifiers and names to antibiotic resistance genes, proteins, and mutations (McArthur et al. 2013). Resistance mechanisms were identified based on the ‘resistance mechanism’ annotation in CARD (v3.2.3; BLASTP e‐value ≤ 1 × 10^−5^).

### 2.3 Social Network Data Processing

Grooming behavior serves to strengthen affiliative relationships among individuals. In nonhuman primate groups, grooming behaviors are recognized as key indicators of such social bonds (Arseneau‐Robar et al. 2018). The Grooming Index (GI) was calculated based on grooming duration between individuals, following the method described by Hashimoto et al. (1996), using the formula:

$$\mathrm{GI}=\frac{\mathrm{fA}\left(B\right)+\mathrm{fB}\left(A\right)}{F\left(A\right)+F\left(B\right)}$$

Here, fA(B) denotes the total grooming time involving individual B (either as groomer or recipient) during focal sampling of individual A; fB(A) represents the total grooming time involving individual A during focal sampling of individual B. F(A) and F(B) refer to the total observation durations for individuals A and B, respectively. Using R software (version 4.4.3), the host Eigenvector Centrality coefficient was computed for all 21 individuals based on the resulting GI matrix. The calculated centrality values are provided in Table S2.

Host Eigenvector Centrality coefficient reflects the relative importance of an individual within a social network: higher values indicate more frequent social interactions, stronger affiliative tendencies, or a more central position in the group structure (Peng 2025; Zhang 2013). Accordingly, to examine the potential link between social network structure and ARG profiles, we incorporated host Eigenvector Centrality coefficient as a key social metric in our analysis.

### 2.4 Data Analysis

All statistical analyses in this study were conducted in R (version 4.3.3) alongside specialized bioinformatics tools. Microbial relative abundance was calculated using TPM (transcripts per million), while ARGs relative abundance was quantified using PPM (parts per million). We adopted ≥ 1% mean relative abundance as the cutoff for high‐abundance ARGs to minimize rare‐sequence noise while retaining potential functional genes. Alpha diversity of gut bacterial community was assessed using the Sobs index (observed species richness) and the Shannon index (community diversity). For beta diversity, a Bray–Curtis distance matrix was generated with QIIME (version 1.9.1), which was subsequently used in further comparative analyses.

This study employs a mixed‐method approach that integrates both hypothesis‐driven and exploratory analyses. Generalized Linear Models (GLMs) were applied to assess the effects of host age and host Eigenvector Centrality coefficient on the diversity of ARGs in the gut microbiome of Tibetan macaques, using the Sobs index, Shannon index, and beta diversity indices as response variables; all three diversity metrics were tested. Predictors included host age, sex, and the Eigenvector Centrality coefficient of the grooming network. Age and Eigenvector Centrality were *z*‐standardized (mean = 0, SD = 1) before entry into the GLMs; sex was treated as a binary factor. To identify differentially abundant microbes and ARGs between sex groups, the Wilcoxon rank‐sum test was employed, with subsequent false discovery rate (FDR) correction using the Benjamini–Hochberg procedure. Associations involving host age and the Eigenvector Centrality coefficient were evaluated using Spearman's rank correlation test.

Data visualization—including stacked bar plots, principal coordinates analysis (PCoA), and heatmaps—was performed using the ggplot2 package in R (version 4.4.3). The abundance data of bacteria and ARGs were normalized to achieve relative abundance. Co‐occurrence networks were constructed as an exploratory analysis to visualize potential interaction patterns between gut bacteria and ARGs. Networks were based on Spearman correlation coefficients (FDR‐corrected) derived from the relative abundances of bacterial taxa and ARGs, with edges and nodes defined by significant correlations. Importantly, this network analysis is exploratory in nature and intended to identify candidate relationships and patterns for future hypothesis‐driven testing rather than to confirm specific a priori hypotheses about bacteria‐ARG associations. Network topology was visualized using Gephi version 0.10.1. A statistical significance threshold of *p* < 0.05 was applied throughout the analysis.

## 3 Results

### 3.1 Composition of Antibiotic Resistance Genes in the Gut Micromicrobiome of Tibetan Macaques

**FIGURE 2:**
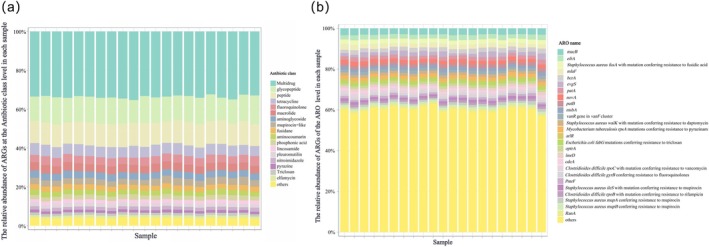
Composition of antibiotic resistance genes (ARGs) in the gut microbiome of Tibetan macaques (total *n* = 21). (a) Relative abundance of different antibiotic resistance classes across samples. (b) Relative abundance of individual ARGs at the ARO level.

In total, 1027 distinct ARGs were identified, distributed across 35 resistance categories. Multidrug resistance genes were the most abundant, representing 33.72% ± 0.75% of the total ARGs, followed by glycopeptide resistance genes (13.17% ± 0.76%) and peptide resistance genes (11.71% ± 0.41%) (Figure 2a). At the gene level based on the ARO, 26 high‐abundance ARGs were defined based on a mean relative abundance threshold of ≥ 1%. These included *macB* (3.06% ± 0.19%), *efrA* (2.32% ± 0.22%), and *Staphylococcus aureus fusA* with mutation conferring resistance to fusidic acid (fusA (mut), 2.18% ± 0.09%) (Figure 2b), among others. In terms of resistance mechanisms, the majority of ARGs were associated with antibiotic target alteration (47.53% ± 1.60%) and antibiotic efflux (39.21% ± 1.73%).

### 3.2 Effects of Sex, Age, and Social Network on ARG Composition and Diversity

**FIGURE 3:**
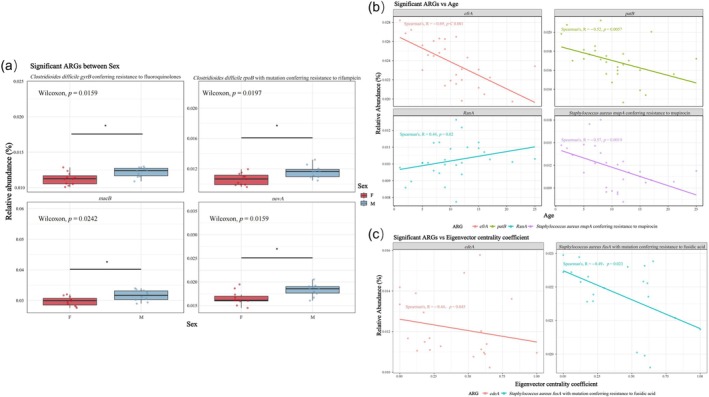
High‐abundance ARGs significantly associated with individual factors (total *n* = 21, including female *n* = 11 and male *n* = 10). (a) Relative abundance of ARGs that differed significantly between sexes. (b) ARGs showing significant correlations with age. (c) ARGs significantly correlated with Eigenvector Centrality coefficient.

This study examined the associations between host sex, age, and Eigenvector Centrality coefficient with the 26 identified high‐abundance ARGs. Differential abundance analysis between male and female groups, conducted using the Wilcoxon rank‐sum test with BH‐FDR correction, revealed four ARGs with statistically significant differences (*p* < 0.05; Figure 3a). These included *Clostridioides difficile gryB* conferring fluoroquinolone resistance (*p* = 0.0159), *Clostridioides difficile rpoB* with mutation conferring rifampicin resistance (*p* = 0.0197), *macB* (*p* = 0.0242), and *novA* (*p* = 0.0159). The relative abundances of these four ARGs in males were significantly higher than those in females.

Spearman's rank correlation analysis between ARG relative abundance and age identified four genes significantly correlated with age (*p* < 0.05; Figure 3b). Three ARGs exhibited a negative correlation: *efrA* (*R* = −0.69, *p* = 7.1 × 10^−5^), *patB* (*R* = −0.52, *p* = 0.0057), and *Staphylococcus aureus mupA* conferring mupirocin resistance (*R* = −0.57, *p* = 0.0019). Only *RanA* showed a significant positive correlation with age (*R* = 0.44, *p* = 0.02).

Similarly, correlation analysis between ARG abundance and host Eigenvector Centrality coefficient identified two genes with significant negative correlations (Figure 3c): *cdeA* (*R* = −0.44, *p* = 0.045) and *Staphylococcus aureus fusA* with mutation conferring fusidic acid resistance (*R* = −0.49, *p* = 0.023).

GLM analyses revealed that no significant associations were observed between the diversity of ARGs in the gut microbiome of Tibetan macaques and the factors of host age, sex, or Eigenvector Centrality coefficient, based on metrics including the Sobs index, Shannon index, and beta diversity (all *p* > 0.05) (Table S3).

### 3.3 Associations Between Gut Bacteria and ARGs

**FIGURE 4:**
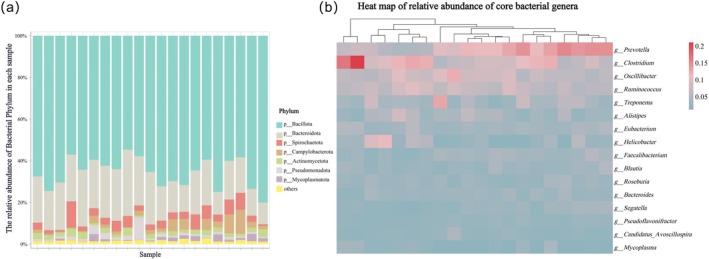
Composition of the gut bacterial community in Tibetan macaques (total *n* = 21). (a) Relative abundance of bacterial taxa at the phylum level across samples. (b) Distribution of core bacterial genera in each sample.

The gut bacterial community of Tibetan macaques was dominated by two phyla: Bacillota (65.82% ± 7.04%) and Bacteroidota (20.23% ± 5.66%) (Figure 4a). Core bacterial genera were defined as those detected in over 90% of fecal samples with a mean relative abundance greater than 1%. Sixteen such core genera were identified: *Prevotella*, *Clostridium*, *Oscillibacter*, *Ruminococcus*, *Treponema*, *Alistipes*, *Eubacterium*, *Helicobacter*, *Faecalibacterium*, *Blautia*, *Roseburia*, *Bacteroides*, *Segatella*, *Pseudoflavonifractor*, Candidatus *Avoscillospira*, and *Mycoplasma*. Among these, *Prevotella* (9.62% ± 3.88%), *Clostridium* (9.34% ± 4.03%), and *Oscillibacter* (6.65% ± 1.90%) were the most abundant (Figure 4b).

**FIGURE 5:**
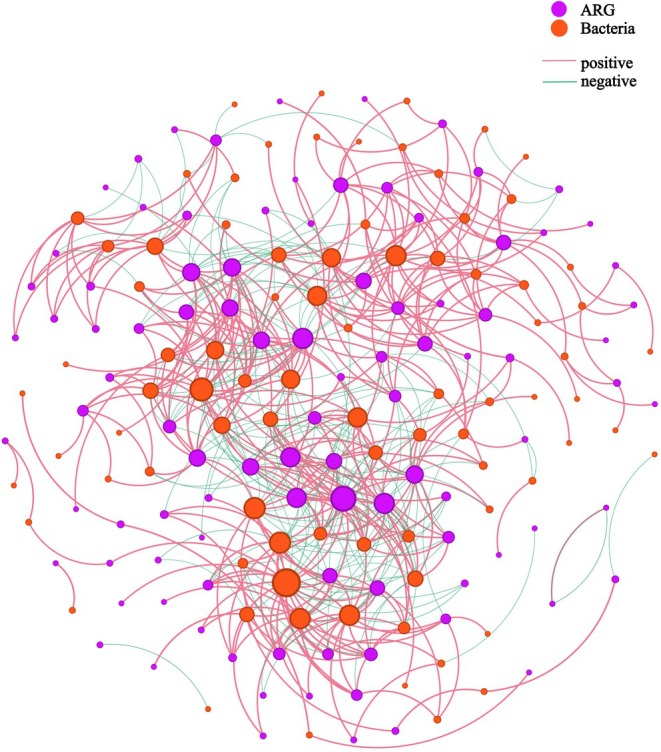
Co‐occurrence network between gut bacteria and ARGs in Tibetan macaques. The network was constructed based on bacterial genera and ARGs with a mean relative abundance > 0.001 (data were normalized). Connections represent significant correlations (|*R*| > 0.7, *p* < 0.01), excluding correlations within the same kingdom. Node size is proportional to the degree of connectivity.

As an exploratory approach, co‐occurrence network analysis at the genus level revealed extensive potential interactions between bacteria and ARGs in the gut microbiome of Tibetan macaques (Figure 5). Within the network, ARGs constituted 52.79% of the nodes, while bacterial taxa accounted for 47.21%. Of the total interactions, 67.76% were positive correlations and 32.24% were negative. Based on node degree centrality, the core nodes in the ARG‐bacteria network were *Thomasclavelia* (degree = 50), the *vanZ* gene in the *vanF* cluster (degree = 44), and *Anaerobutyricum* (degree = 40). Among the top 10 nodes ranked by degree, only three were ARGs: the *vanZ* gene (degree = 44), *rosB* (degree = 34), and the *vanS* gene in the *vanP* cluster (degree = 34). Notably, both the *vanZ* and *vanS* genes are involved in glycopeptide resistance, whereas *rosB* confers resistance to peptide antibiotics.

No significant correlation was observed between bacterial diversity and the abundance of ARGs in this study. Using consistent analytical approaches, we further evaluated the effects of host sex, age, and Eigenvector Centrality coefficient on both alpha and beta diversity of the gut bacteria. The results indicated that neither host age nor host Eigenvector Centrality coefficient had a significant influence on bacterial diversity. In line with the ARG analysis, we employed the Wilcoxon rank‐sum test with BH‐FDR correction and Spearman's rank correlation to identify core bacterial genera associated with host sex, age, and Eigenvector Centrality coefficient. Four core genera showed significant differences between sexes: *Roseburia* (*p* = 0.0159), *Oscillibacter* (*p* = 0.0197), Candidatus *Avoscillospira* (*p* = 0.0242), and *Ruminococcus* (*p* = 0.0295) (Figure S1a). Five core genera were significantly correlated with age: *Alistipes* (*R* = 0.55, *p* = 0.003), *Bacteroides* (*R* = 0.47, *p* = 0.013), *Clostridium* (*R* = −0.60, *p* = 0.00093), *Mycoplasma* (*R* = −0.55, *p* = 0.00293), and *Roseburia* (*R* = 0.44, *p* = 0.022) (Figure S1b). Two genera were significantly correlated with host Eigenvector Centrality coefficient: *Alistipes* (*R* = 0.44, *p* = 0.046) and *Eubacterium* (*R* = −0.46, *p* = 0.038) (Figure S1c).

We then examined whether the core genera and high‐abundance ARGs that were significantly associated with host sex, age, or Eigenvector Centrality coefficient also appeared as correlated pairs in the co‐occurrence network. No significant correlations were found between bacteria and ARGs that differed by host sex. However, among the five bacteria and four ARGs correlated with host age, four significant bacteria–ARG pairs were identified. Similarly, among the two bacteria and two ARGs correlated with host Eigenvector Centrality coefficient, one significant correlation pair was detected (Table 1).

**TABLE 1:** Bacterium–ARG pairs with shared significant correlations to age or Eigenvector Centrality coefficient.

	Source	Target	Weight	*p*	Direction
Age	*Bacteroides*	*patB*	−0.844	1.51E‐06	Negative
	*Bacteroides*	*Staphylococcus aureus mupA* conferring resistance to mupirocin	−0.743	1.15E‐04	Negative
	*Clostridium*	*Staphylococcus aureus mupA* conferring resistance to mupirocin	0.76	6.46E‐05	Positive
	*Mycoplasma*	*Staphylococcus aureus mupA* conferring resistance to mupirocin	0.914	6.84E‐09	Positive
Eigenvector Centrality coefficient	*Eubacterium*	*patB*	0.701	3.97E‐04	Positive

## 4 Discussion

This study focuses on the bacterial communities and ARGs in the gut microbiome of wild Tibetan macaques living in Mount Huangshan. The findings reveal that the intestinal ARGs in these macaques are predominantly multidrug, glycopeptide, and peptide resistance genes. This pattern is consistent with a recent study on ARGs in Tibetan macaques from the Mabian Nature Reserve in Sichuan (Wu 2024), yet differs significantly from results reported in most other NHP studies, where tetracycline resistance genes are dominant (Huang et al. 2023; Yan et al. 2022; Campbell et al. 2020). Previous research has indicated that tetracycline antibiotics are among the most widely used and heavily applied classes in agriculture (Van Boeckel et al. 2015). The dominant tetracycline resistance genes detected in primates show high overlap with those prevalent in agricultural animals (Lim et al. 2020), which may be linked to the historical use of tetracyclines in agricultural environments (Huang et al. 2023). In contrast, the enrichment of multidrug resistance genes is the combined outcome of complex environmental pressures and diverse human activities (Mazhar et al. 2021; Li, Yang, et al. 2015; Zhu et al. 2017). Although Mabian has not developed tourism, Tibetan macaques there are frequently exposed to complex anthropogenic pollution through crop foraging, scavenging household waste, and close contact with residents during livelihood activities such as bamboo‐shoot harvesting (Wu 2024). Similarly, the habitat of the Tibetan macaques in Huangshan is subject to high‐intensity and specific human‐induced pressures, including feeding by tourists and surrounding farmers, discarded food packaging, and potential human fecal contamination. These findings collectively suggest that the distinct multidrug resistome in the gut of the Huangshan macaques is primarily driven by exposure to complex domestic waste resulting from the overlap of their habitat with human residential areas, rather than traditional agricultural antibiotic pollution.

In animal and human studies, factors such as age and sex have been shown to significantly influence the distribution of ARGs. For instance, the composition of rumen ARGs in goat kids changes with age, and their richness declines as age increases (Chai et al. 2024). Similarly, the relative abundance of ARGs in the gut of juvenile giant pandas is higher than that in adults (Zhu et al. 2021). In human populations, women in high‐income countries exhibit significantly greater ARG diversity than men (Salehi et al. 2025). However, in the gut microbiome of Tibetan macaques examined in this study, no significant correlations were observed between ARG overall diversity and host sex, age, or eigenvector centrality. This suggests that these factors may not be key determinants of ARG diversity in wild Tibetan macaques, though the specific reasons for these results remain unclear based on the currently available data. Future studies should expand the sample size, conduct longitudinal tracking, and integrate observations of host behavior with measurements of environmental factors to further elucidate the dynamics of intestinal ARG diversity in Tibetan macaques and its relationship with socio‐ecological variables.

It is noteworthy that, although no significant correlation was observed at the level of overall diversity, the distribution of core ARGs was still influenced by host sex, age, and social structural characteristics. For instance, we found that four highly abundant ARGs, including *Clostridioides difficile gryB* conferring fluoroquinolone resistance, *Clostridioides difficile rpoB* with mutation conferring rifampicin resistance, *macB*, and *novA*, were significantly enriched in male individuals. This result suggests that sex may differentially shape the antimicrobial resistance profile in the gut of Tibetan macaques by modulating the abundance of key ARGs. Among these, *gyrB* and *rpoB* directly mediate resistance to important clinical antibiotics (Hooper and Jacoby 2015; Goldstein 2014), and their enrichment can be regarded as direct molecular evidence of clinical resistance spillover into the environment. *macB* is often located on mobile genetic elements, indicating potential horizontal gene transfer and multidrug resistance risk (Partridge et al. 2018). *novA* may serve as a relic or co‐selection marker persisting in the resistance gene reservoir. The co‐occurrence of these ARGs in male gut suggests that their gut may constitute a microenvironment harboring both historic and emerging resistance mechanisms, with potential for gene flow. This sexual dimorphism is likely related to the behavioral ecology and physiological traits of male Tibetan macaques. Behaviorally, males typically possess larger home ranges and higher mobility (Bates and Byrne 2009) and more frequently explore and enter areas of intense human activity (e.g., tourist zones, waste sites) (Haux et al. 2023; Ji et al. 2010), thereby increasing their exposure to environmental ARG contamination (Marty et al. 2020; Balasubramaniam et al. 2020). Thus, male individuals may act as key bridges connecting anthropogenic pollution sources with the microbial reservoir within the troop. Second, physiological differences may also play a supporting role: sex hormones (such as testosterone) can influence the gut microenvironment by modulating immune responses (Buendía‐González and Legorreta‐Herrera 2022) and bile acid signaling pathways (Duan et al. 2024; Yoon and Kim 2021; Maffei et al. 2022), potentially altering the colonization efficiency of resistant bacteria or plasmid transfer dynamics, thereby giving the male gut a different propensity for carrying or maintaining specific ARGs. In summary, this study indicates that male Tibetan macaques may play the role of reservoirs and diffusion hubs for antibiotic resistance genes within the population. Their ecological function within the One Health framework warrants further attention.

Furthermore, this study revealed that host age was negatively correlated with the relative abundance of four ARGs: *efrA*, *patB*, and *Staphylococcus aureus mupA* conferring mupirocin, while it showed a significant positive correlation with *RanA*. Part of our findings aligns with results from other studies. For example, research on ARGs in the gut of giant pandas also reported a decrease in the relative abundance of the *efrA* gene in older age groups (Mustafa et al. 2021). Such age‐related changes may be driven by multiple factors. On one hand, immune senescence, accompanied by declining immune function, may reshape the structure of the gut microbiota and its associated resistome (Theodorakis et al. 2024). On the other hand, age‐related shifts in microbial composition (such as changes in taxa like Bacteroidaceae and Prevotellaceae) may directly affect the enrichment of specific ARGs. Similar phenomena have been reported in studies on the rumen resistome of goats (Chai et al. 2024). Meanwhile, this study also found that the abundance of *cdeA* and *Staphylococcus aureus fusA* with mutation conferring fusidic acid were significantly negatively correlated with the host Eigenvector Centrality coefficient. This suggests that these ARGs tend to be enriched more in individuals at the periphery of the social network rather than in those occupying central positions. This distribution pattern may be related to the metabolic cost associated with ARGs. Both *cdeA*, which encodes a multidrug efflux pump, and *fusA*, which mediates fusidic acid resistance, entail substantial metabolic costs (Piddock 2006; Andersson and Hughes 2010; Besier et al. 2005). Core individuals, in order to maintain their key ecological functions, may tend to suppress the accumulation of such high‐cost resistance genes. In contrast, peripheral hosts face stronger environmental pressures and competition, which may limit their ecological opportunities to move toward central positions within the network.

The study revealed that the intestinal bacterial community of Tibetan macaques was dominated by the phyla Bacillota and Bacteroidota, with several core genera playing key roles in maintaining gut microecological homeostasis and function. Co‐occurrence network analysis at the genus level further indicated widespread positive and negative correlations between bacteria and ARGs, suggesting complex coevolutionary and interactive mechanisms between them. Several core nodes, such as the genus *Thomasclaveria* and the *vanZ* gene within the *vanF* cluster, played improtant roles in the ARG—Bacteria co‐occurrence network, potentially serving as hubs linking bacteria and ARGs and thereby facilitating the dissemination and spread of resistance genes within the microbial community. Moreover, no significant correlation was observed between bacterial diversity and the abundance of ARGs. This finding differs from some existing studies: for example, a metagenomic study on giant pandas reported a clear negative correlation between bacterial diversity and ARG abundance (Zhu et al. 2021), and other research has also suggested that higher bacterial diversity may suppress the spread of resistance genes through niche competition (Van Elsas et al. 2012; Kennedy et al. 2002; Gionchetta et al. 2024). Such inconsistency indicates that the relationship between bacterial diversity and ARG abundance is not constant but is coregulated by multiple factors, such as environmental selection pressure, methodological differences, and cross‐kingdom biotic interactions. Additionally, single‐time‐point sampling may fail to capture the dynamic nature of the diversity‐resistance relationship. Future studies should employ longitudinal cohort designs, single‐strain‐resolution sequencing, and in vitro gut models to further validate the robustness of this negative result.

Although several core bacterial genera showed significant associations with factors such as host sex, age, and Eigenvector Centrality, and certain high‐abundance ARGs were linked to host sex, age, and Eigenvector Centrality, no coordinated variation was identified between bacteria and ARGs specifically related to host sex or Eigenvector Centrality. In contrast, multiple significant correlations were observed between host age‐associated bacteria and ARGs. Notably, a network of associations was identified between ARGs (including *Staphylococcus aureus mupA* and *patB*) and bacterial genera such as *Bacteroides*, *Clostridium*, and *Mycoplasma*, which themselves showed host age‐related patterns. Collectively, these results suggest that host age may be an important factor modulating the correlation between specific bacterial genera and ARGs in this monkey population. In nonhuman primates, *Bacteroides* is generally recognized as a marker of gut health (Sun et al. 2023). Host age‐related shifts in diet and lifestyle may promote the proliferation of *Bacteroides*, whose diverse metabolic outputs and immunomodulatory properties may in turn suppress the growth of *Staphylococcus aureus carrying mupA* and other bacteria hosting *patB*. This negative correlation was more pronounced in older individuals, suggesting that *Bacteroides* may contribute to maintaining gut homeostasis and restricting the expansion of resistant bacteria in aged hosts. In contrast, *Clostridium*, known for its versatile metabolic activity in the gut (Cazzaniga et al. 2024), may produce specific nutrients or metabolites that support the growth of *Staphylococcus aureus* . Meanwhile, *Mycoplasma* may facilitate the horizontal transfer of resistance genes to other bacteria (Citti et al. 2018; Pereyre and Tardy 2021), thereby promoting the dissemination of ARGs. These positive correlations were more evident in younger macaques, indicating that *Clostridium* and *Mycoplasma* may play more prominent roles in the spread of resistance genes during early life stages.

Collectively, these findings highlight host age as an important factor influencing the coordinated variation between gut microbes and ARGs in Tibetan macaques. By modulating the abundance of specific core genera, host age may be indirectly linked to changes in ARG prevalence—a pattern that may have been shaped by the combined effects of host physiology and ecological factors over long‐term evolution.

## 5 Conclusion

This study presents the first in‐depth metagenomic analysis of the bacterial community and ARGs in the gut microbiome of Tibetan macaques living in Mount Huangshan. By systematically characterizing the composition of ARGs, evaluating the influence of individual factors on ARG diversity and high‐abundance genes, and exploring the interrelationships between gut microbes and ARGs, this work reveals the complexity of the gut resistome and its drivers in this primate species. The results demonstrate that the gut microbiome of Tibetan macaques harbors a diverse repertoire of ARGs, dominated by multidrug, glycopeptide, and peptide resistance types—a profile distinct from that reported in other nonhuman primates and indicative of a heightened propensity for accumulating multidrug resistance genes. The primary resistance mechanisms identified were antibiotic target alteration and antibiotic efflux. Although host age, sex, and Eigenvector Centrality coefficient did not significantly influence overall ARG diversity, they were significantly correlated with the relative abundance of several high‐abundance ARGs. Notably, the influence of social network centrality was restricted to only two ARGs (*cdeA* and *fusA(mut)*), whereas age‐related effects were more widespread and robust. These findings suggest that host age is a stronger and more pervasive driver of ARG profile variation than social network position, with social interactions potentially playing a secondary role in influencing the abundance of these specific resistance genes via selective transmission or shared environmental exposure. Furthermore, extensive bacteria–ARG interactions were observed, with host age emerging as a key driver of their covariation.

These findings elucidate the composition and drivers of the gut resistome in wild primates, establishing a scientific foundation for monitoring, risk assessment, and intervention strategies targeting antimicrobial resistance in wildlife. Future efforts may focus on the specific indicators identified here: (1) host‐linked ARG biomarkers (e.g., *gryB*, *rpoB*, *macB*, and *novA* for host sex; *efrA*, *patB*, *mupA*, and *RanA* for host age; *cdeA* and *fusA (mut)* for social centrality); (2) environmental exposure indicators, such as human‐associated waste and the abundance of ARGs and mobile genetic elements in shared soil and water; (3) host behavioral metrics, particularly eigenvector centrality; and (4) accompanying shifts in core gut microbiota (e.g., *Bacteroides*, *Clostridium*, *Roseburia*) that covary with the resistome. Longitudinal tracking of these integrated indicators would advance from descriptive profiling toward a mechanistic understanding of resistome dynamics, ultimately supporting targeted risk assessment and mitigation of antimicrobial resistance in wildlife. Further studies across broader ecological gradients are needed to validate these indicators and clarify the complex interplay between host physiology, ecology, and the resistome.

This study was conducted on a troop accustomed to long‐term supplemental feeding. Although maize accounted for roughly one‐third of the diet and human contact was minimized, we cannot fully rule out a diluting or amplifying effect of provisioning on the gut microbiota and ARG background. Caution is warranted when extrapolating these findings to nonprovisioned populations. Meanwhile, because of the troop's small size and the logistical constraints of fieldwork, future studies should enlarge sample sizes across multiple Huangshan troops or other populations to test the generality of our conclusions.

## Author Contributions

**Yue Ling:** conceptualization (equal), data curation (equal), formal analysis (equal), investigation (equal), methodology (equal), software (equal), validation (equal), visualization (equal), writing – original draft (equal), writing – review and editing (equal). **Dong‐Xin Yang:** data curation (equal), formal analysis (equal), investigation (equal), methodology (equal), software (equal). **Ying‐Na Xia:** data curation (equal), formal analysis (equal), investigation (equal), resources (equal). **Chuan‐Peng Bao:** conceptualization (equal), data curation (equal), formal analysis (equal), investigation (equal). **Fan Zhang:** data curation (equal), investigation (equal), resources (equal). **Xiao‐Juan Xu:** conceptualization (equal), data curation (equal), writing – review and editing (equal). **Bing‐Hua Sun:** conceptualization (equal), data curation (equal), funding acquisition (lead), project administration (equal), resources (equal), supervision (equal), writing – review and editing (equal).

## Funding

This research was supported by the National Natural Science Foundation of China (grant numbers: 32171488, 32300400).

## Conflicts of Interest

The authors declare no conflicts of interest.

## Supporting information


**Figure S1:** Core bacterias significantly associated with individual factors (total *n* = 21, including female *n* = 11 and male *n* = 10). (a) Relative abundance of bacterias that differed significantly between sexes. (b) Bacterias showing significant correlations with age. (c) Bacterias showing significant correlations with Eigenvector Centrality coefficient.

**Table S1:** Fecal sample collection information form.

**Table S2:** The Eigenvector centrality coefficient of each individual.

**Table S3:** The influence of gender, age, and eigenvector centrality coefficient on diversity index.

## Data Availability

The Raw Data supporting the findings of this study are available in the NCBI database (https://www.ncbi.nlm.nih.gov/). The data can be accessed at https://www.ncbi.nlm.nih.gov/bioproject/PRJNA1353630. Additional data related to this study may be requested from the corresponding author upon reasonable request.
