# Supplementary figures and images for: Effects of Age, Sex, and Social Network on Antibiotic Resistance Genes in the Gut Microbiome of Tibetan Macaques (Macaca thibetana)
Source: Ecol Evol. 2026 Feb 20;16(2):e73137. doi: 10.1002/ece3.73137 (PMC12928124; doi:10.1002/ece3.73137)

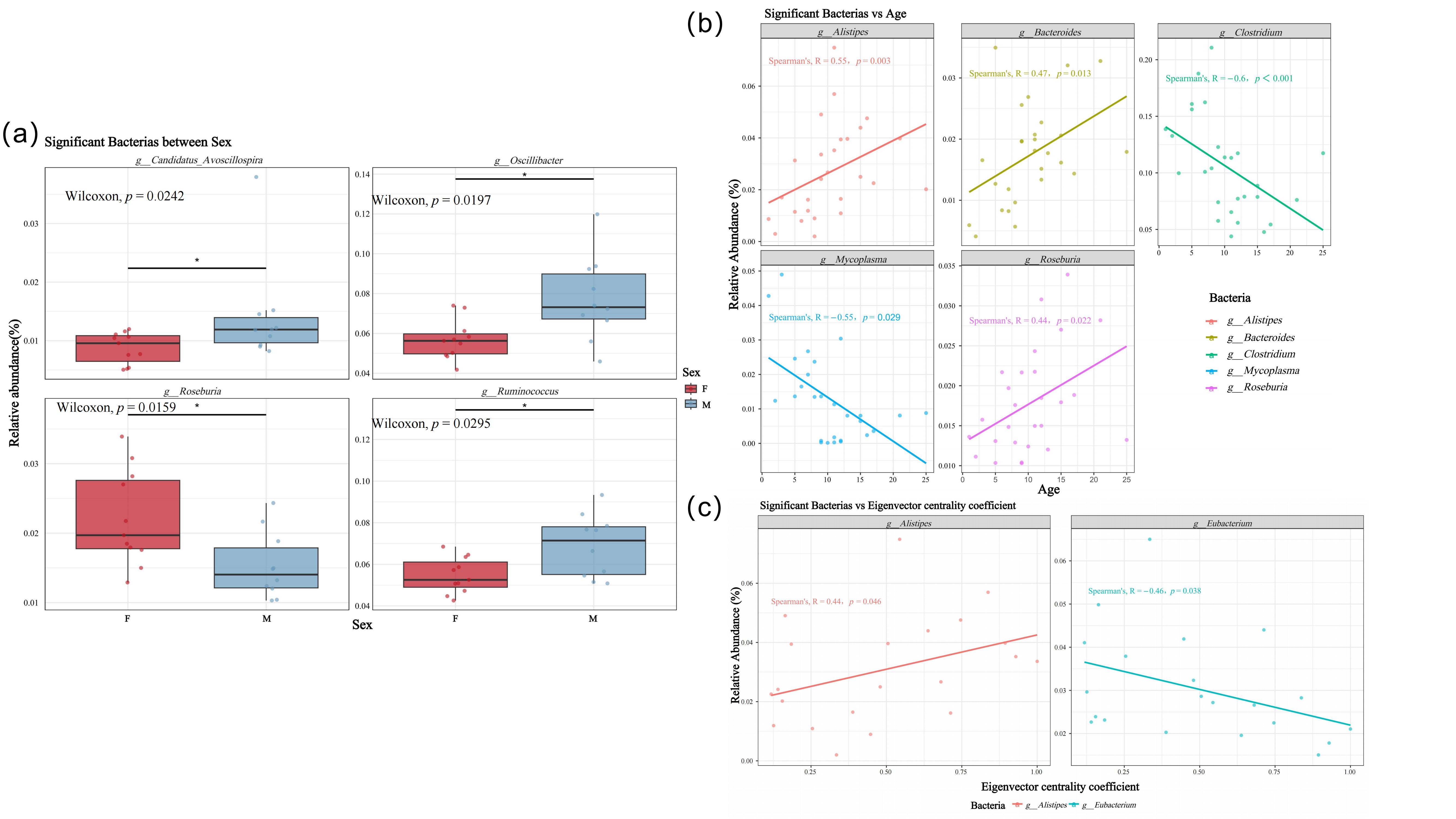

Supplement: Supplementary file 1 — Figure S1: Core bacterias significantly associated with individual factors (total n = 21, including female n = 11 and male n = 10). (a) Relative abundance of bacterias that differed significantly between sexes. (b) Bacterias showing significant correlations with age. (c) Bacterias showing significant correlations with Eigenvector Centrality coefficient. [file ECE3-16-e73137-s001.jpg]
