# Supplementary material for: Effects of Age, Sex, and Social Network on Antibiotic Resistance Genes in the Gut Microbiome of Tibetan Macaques (Macaca thibetana)
Source: Ecol Evol. 2026 Feb 20;16(2):e73137. doi: 10.1002/ece3.73137 (PMC12928124; doi:10.1002/ece3.73137)
Supplement: Supplementary file 2 — Table S1: Fecal sample collection information form. [file ECE3-16-e73137-s003.pdf]

Table S1 Fecal sample collection information form

SampleID	Individual name	Sex	Age	Sampling time
S1	YL	Male	10	2024.8.17
S2	TQS	Male	9	2024.8.7
S3	TQ	Male	13	2024.8.17
S4	NM	Male	11	2024.8.18
S5	DZ	Male	9	2024.8.15
S6	YCH	Female	12	2024.8.15
S7	TXH	Female	15	2024.8.18
S8	TQL	Female	11	2024.8.13
S9	TXX	Female	16	2024.8.13
S10	THX	Female	12	2024.8.13
S11	WM	Male	17	2024.8.27
S12	YXK	Male	11	2024.7.24
S13	LB	Male	9	2024.7.16
S14	BM	Male	25	2024.8.13
S15	YXM	Male	7	2024.8.11
S16	YCL	Female	12	2024.8.3
S17	TQG	Female	7	2024.8.16
S18	TH	Female	21	2024.7.9
S19	THY	Female	15	2024.8.10
S20	TQY	Female	8	2024.8.8
S21	TFH	Female	8	2024.8.26
